# Supplementary material for: Effect of Anti-Interleukin-6 Agents on Psychopathology in a Sample of Patients with Post-COVID-19 Syndrome: An Observational Study
Source: Brain Sci. 2024 Jan 3;14(1):47. doi: 10.3390/brainsci14010047 (PMC10813526; doi:10.3390/brainsci14010047)
Supplement: Supplementary file 1 [file brainsci-14-00047-s001.zip › brainsci-2778481-supplementary.pdf]

brain sciences

Supplement

Table S1:………………………………………………………………………………………….Pag 2
Psychopathological scales: ……………………………………………………………………….Pag 3

Table S1. Pearson correlations among psychopathological scales.

	BPRS Total	HAMA Total	HAM-D Total	MRS Total	KMRSD Total	CAPS Total	CD-RISC Total	DERS Total	SHAPS Total	PSQI Total	BHS Total
BPRS Total	1	.542	.616	.355	.446	.491	-.170	.201	.106	.261	.016
HAM-A Total	.542	1	.866	.432	.530	.738	-.255	.223	.115	.167	.030
HAM-D Total	.616	.866	1	.491	.600	.730	-.231	.229	.149	.160	.001
MRS Total	.355	.432	.491	1	.564	.512	-.077	.112	.000	.057	-.030
KMRSD Total	.446	.530	.600	.564	1	.611	-.137	.193	-.025	.112	-.008
CAPS Total	.491	.738	.730	.512	.611	1	-.196	.220	.030	.146	.039
CD-RISC Total	-.170	-.255	-.231	-.077	-.137	-.196	1	-.264	-.066	-.131	.186
DERS Total	.201	.223	.229	.112	.193	.220	-.264	1	.182	.118	.116
SHPS_TOT Total	.106	.115	.149	.000	-.025	.030	-.066	.182	1	.084	.150
PSQI_TOT Total	.261	.167	.160	.057	.112	.146	-.131	.118	.084	1	.017
BHS_TOT Total	.016	.030	.001	-.030	-.008	.039	.186	.116	.150	.017	1

Legend: BHS, Beck Hopelessness Scale**; BPRS,** Brief-Psychiatric Rating Scale; **CAPS,** Clinician-Administered PTSD Scale**, CD-RISC,** Connor-Davidson Resilience Scale; **Ctrl,** patients who did not receive anti-interleukin-6-receptor treatment; **DERS,** Difficulties in Emotion Regulation scale; **HAM-A,** Hamilton Anxiety rating scale; **HAM-D,** Hamilton rating scale for depression; **KMDRS,** Koukopoulos Mixed Depression Rating Scale; **PSQI,** Pittsburgh Sleep Quality Index; **SAR,** patients receiving sarilumab during hospitalization; **SD,** standard deviation; **SHAPS,** Snaith-Hamilton Pleasure Scale; **SpO2,** peripheral capillary oxygen saturation; **TOC**, patients receiving Tocilizumab during hospitalization.

CD-RISC - Scala di Connor-Davidson

Istruzioni: per cortesia, indicate quanto siete d'accordo con le seguenti affermazioni in base a quando si sono verificate il mese scorso. Se una di queste situazioni non si e' verificata di recente, rispondete in base a cio' che avreste provato.

	MAI	QUASI MAI	A VOLTE	SPESSO	MOLTO SPESSO
1. Sono in grado di adattarmi ai cambiamenti.	○	○	○	○	○
2. Ho almeno una relazione intima e stabile su cui posso contare nei momenti di difficolta'.	○	○	○	○	○
3. Quando non vedo una via d'uscita ai miei problemi, penso che Dio o il destino possono venirmi incontro.	○	○	○	○	○
4. Posso affrontare qualsiasi imprevisto.	○	○	○	○	○
5. I risultati positivi ottenuti in passato mi incoraggiano ad affrontare nuove sfide e difficolta'.	○	○	○	○	○
6. Cerco di vedere il lato ironico dei problemi.	○	○	○	○	○
7. Affrontare eventi stressanti mi rende piu' forte.	○	○	○	○	○
8. Tendo a lasciarmi alle spalle episodi negativi del passato,come i problemi di salute, i danni subiti, o altre avversita'.	○	○	○	○	○
9. Credo che la maggior parte degli eventi, buoni o cattivi, accadono per una precisa ragione.	○	○	○	○	○
10. Mi impegno a dare il meglio di me, indipendentemente dal risultato.	○	○	○	○	○
11. Sono convinto di poter raggiungere i miei obbiettivi, anche in presenza di ostacoli.	○	○	○	○	○
12. Affronto gli ostacoli anche quando sembrano insuperabili.	○	○	○	○	○
13. So a chi rivolgermi per avere un aiuto nei momenti di crisi.	○	○	○	○	○

projectredcap.org REDCap®

14. Quando sono sotto pressione, riesco a rimanere lucido e concentrato su quello che sto facendo.	○	○	○	○	○
15. Preferisco cercare di risolvere i miei problemi da solo, piuttosto che lasciarlo fare agli altri.	○	○	○	○	○
16. Non mi lascio abbattere facilmente dagli insuccessi.	○	○	○	○	○
17. Mi considero una persona decisa nell'affrontare le sfide e le difficolta' della vita.	○	○	○	○	○
18. Se necessario, sono capace di prendere decisioni impopolari o difficili per gli altri.	○	○	○	○	○
19. Sono in grado di sopportare sentimenti spiacevoli e dolorosi, come tristezza, rabbia e paura.	○	○	○	○	○
20. Talvolta per riuscire a cavarsela bisogna fidarsi del proprio istinto ed affidarsi al destino.	○	○	○	○	○
21. Sono convinto che nella vita ci sia un senso.	○	○	○	○	○
22. Sento di avere il controllo della mia vita.	○	○	○	○	○
23. Amo le sfide.	○	○	○	○	○
24. Mi impegno per raggiungere i miei obiettivi, senza preoccuparmi degli ostacoli che incontro.	○	○	○	○	○
25. Sono fiero di quello che ho realizzato.	○	○	○	○	○

DERS - Difficulties in Emotion Regulation Scale

Utilizzando la seguente scala di valori, le chiediamo di segnare quanto spesso le seguenti affermazioni possono essere applicate alla sua esperienza, cerchiando il numero appropriato a fianco di ogni item.

	Quasi mai (0-10%)	a volte (11-35%)	circa la meta' delle volte (36-65%)	molte volte (66-90%)	quasi sempre (91-100%)
1. Sono sereno riguardo a cio' che provo	○	○	○	○	○
2. Presto attenzione a come mi sento	○	○	○	○	○
3. Vivo le mie emozioni come travolgenti e fuori dal controllo	○	○	○	○	○
4. Non ho idea di come mi sento	○	○	○	○	○
5. Ho difficolta' a dare un senso a cio' che provo	○	○	○	○	○
6. Sono attento alle mie sensazioni	○	○	○	○	○
7. So esattamente come mi sento	○	○	○	○	○
8. Cerco di capire come mi sento	○	○	○	○	○
9. Sono confuso riguardo a cio' che provo	○	○	○	○	○
10. Quando sono turbato, riconosco le mie emozioni	○	○	○	○	○
11. Quando sono turbato, mi arrabbio con me stesso perche' mi sento in quel modo	○	○	○	○	○
12. Quando sono turbato, mi imbarazza sentirmi in quel modo	○	○	○	○	○
13. Quando sono turbato, ho delle difficolta' a completare il mio lavoro	○	○	○	○	○
14. Quando sono turbato, perdo il controllo	○	○	○	○	○
15. Quando sono turbato, credo che rimarro' in quello stato per molto tempo	○	○	○	○	○
16. Quando sono turbato, credo che finiro' per sentirmi molto depresso	○	○	○	○	○

17. Quando sono turbato, credo che i miei sentimenti siano validi e importanti
18. Quando sono turbato, faccio fatica a focalizzarmi su altre cose
19. Quando sono turbato, mi sento senza controllo
20. Quando sono turbato, posso comunque finire le cose che devo fare
21. Quando sono turbato, mi vergogno di come mi sento
22. Quando sono turbato, so che posso trovare un modo per sentirmi meglio
23. Quando sono turbato, mi sento come se fossi debole
24. Quando sono turbato, sento di potere avere ancora il controllo dei miei comportamenti
25. Quando sono turbato, mi sento in colpa per sentirmi cosi
26. Quando sono turbato, ho delle difficolta' a concentrarmi
27. Quando sono turbato, ho delle difficolta' nel controllare i miei comportamenti
28. Quando sono turbato, credo che non ci sia niente che io possa fare per sentirmi meglio
29. Quando sono turbato, mi irrito con me stesso perche' mi sento in quel modo
30. Quando sono turbato, inizio a sentirmi molto male con me stesso
31. Quando sono turbato, credo che crogiolarmi in questa emozione sia l'unica cosa che io possa fare
32. Quando sono turbato, perdo il controllo sui miei comportamenti

33. Quando sono turbato, faccio fatica a pensare a qualcosa di diverso	○	○	○	○	○
34. Quando sono turbato, mi prendo del tempo per riflettere su quello che sto provando veramente	○	○	○	○	○
35. Quando sono turbato, mi ci vuole molto tempo per sentirmi meglio	○	○	○	○	○
36. Quando sono turbato, sento che le mie emozioni sono travolgenti	○	○	○	○	○

Snaith Hamilton Pleasure Scale

Segnare la casella corrispondente a QUANTO e' IN ACCORDO con ciascuna affermazione riportata, facendo riferimento a quanto ha provato NELLE ULTIME 2 SETTIMANE:

	Prevalentemente d'accordo	Prevalentemente in disaccordo
1) Di solito mi fanno piacere i miei programmi radiofonici o televisivi favoriti	○	○
2) Di solito mi fa piacere stare con i familiari o con gli amici stretti	○	○
3) Di solito sono in grado di gustarmi i miei cibi preferiti	○	○
4) Di solito provo piacere nei miei hobby o passatempi	○	○
5) Di solito mi fa piacere un bagno caldo o una doccia rinfrescante	○	○
6) Di solito mi fa piacere il profumo dei fiori o l'odore di una fresca brezza marina o quello del pane fresco	○	○
7) Di solito mi fa piacere vedere le facce sorridenti	○	○
8) Di solito mi fa piacere apparire elegante quando ho preso cura del mio aspetto	○	○
9) Di solito provo piacere nel leggere un libro, una rivista o un giornale	○	○
10) Di solito provo piacere a bere un caffe', un the o la mia bevanda preferita	○	○
11) Di solito mi danno piacere anche le piccole cose come ad esempio una luminosa giornata di sole	○	○
12) Di solito mi fa piacere vedere un bel paesaggio o una bella veduta	○	○
13) Di solito mi fa piacere aiutare gli altri	○	○

14) Di solito provo piacere quando ricevo lodi dagli altri	○	○
PSQI<br><br>Le seguenti domande si riferiscono alle abitudini di sonno durante l'ultimo mese. Si dovra' indicare la risposta che si riferisce alla maggior parte dei giorni delle notti dell'ultimo mese.<br><br>1) Durante l'ultimo mese a che ora e' andato a letto?	______________	
PSQI<br>2) Durante l'ultimo mese: quanto tempo (espresso in minuti) ha impiegato ad addormentarsi ogni notte?	______________	
PSQI<br>3) Durante l'ultimo mese: a che ora si e' svegliato la mattina?	______________	
PSQI<br>4) Durante l'ultimo mese: quante ore ha dormito realmente la notte (possono essere differenti dal numero di ore passate a letto)?	______________	

PSQI

5) Durante l'ultimo mese, quanto spesso ha avuto problemi a dormire? Perche'?

	Mai durante l'ultimo mese	Meno di una volta a settimana	Una o due volte a settimana	Tre o piu' volte a settimana
a) Non riusciva ad addormentarsi entro 30 minuti da quando era andato a letto?				
b) Si svegliava nel mezzo della notte o presto la mattina				
c) Doveva alzarsi per andare in bagno				
d) Non riusciva a respirare bene				
e) Tossiva o respirava rumorosamente				
f) Sentiva caldo				
g) Sentiva freddo				
h) Faceva brutti sogni				
i) Aveva dolore				
j) Per un'altra o piu' ragioni. Puo' descriverle, indicando anche quanto spesso, ha avuto problemi ha dormire per questa ragione?				

PSQI	Mai durante l'ultimo mese	Meno di una volta a settimana	Una o due volte a settimana	Tre o piu' volte a settimana
6) Durante l'ultimo mese quanto spesso ha assunto farmaci (prescritti o di sua iniziativa) per dormire?	○	○	○	○
7) Durante l'ultimo mese quanto spesso ha avuto problemi a stare sveglio, mentre, per esempio, mangiava o era impiegato in qualche altra attivita?	○	○	○	○
8) Durante l'ultimo mese quanto problematico e' stato per lei mantenere l'entusiasmo per le cose che faceva?	○	○	○	○

PSQI
9) Durante l'ultimo mese come valuterebbe la sua qualita' del sonno?

○ Molto buona ○ Abbastanza buona
○ Abbastanza cattiva ○ Molto cattiva

projectredcap.org REDCap

BHS

Istruzioni: Qui sotto vi sono 20 affermazioni. Risponda se per Lei siano vere o false segnando una croce
(+ o x) nelle caselle corrispondenti. In caso di dubbio, dia la risposta che ritiene piu' vicina a quello che Lei
crede corrisponda meglio a quello che pensa. Abbia cura di segnare uno solo tra Vero e Falso per tutte
le affermazioni.

	Falso	Vero
1) Vedo il futuro con speranza ed entusiasmo	○	○
2) Potrei arrendermi perche' non posso migliorare le cose per me	○	○
3) Quando le cose vanno male, mi consola sapere che non puo' durare cosi in eterno	○	○
4) Non posso immaginare quello che sara' della mia vita tra 10 anni	○	○
5) Ho abbastanza tempo per realizzare le cose che desidero fare di piu'	○	○
6) Nel futuro mi aspetto di riuscire in quello che mi interessa di piu'	○	○
7) Il mio futuro mi sembra buio	○	○
8) Mi aspetto di ottenere dalla vita piu' cose buone rispetto alla persona media	○	○
9) Non mi prendo pause e non c'e' motivo per cui lo debba fare in futuro	○	○
10) Le mie esperienze passate mi hanno preparato bene per il futuro	○	○
11) Se guardo avanti vedo solo situazioni spiacevoli piuttosto che piacevoli	○	○
12) Non mi aspetto di ottenere cio' che voglio veramente	○	○

13) Quando guardo al futuro, mi aspetto di essere piu' felice di adesso	○	○
14)Le cose proprio non vanno come io desidero che vadano	○	○
15) Ho una grossa fede nel futuro	○	○
16) Non ottengo mai cio' che desidero, quindi e' sciocco desiderare alcunche'	○	○
17) e' molto inverosimile che nel futuro io ottenga una vera soddisfazione	○	○
18) Il futuro mi sembra vago e incerto	○	○
19) Posso sperare che arrivino bei tempi, piuttosto che brutti	○	○
20) E' inutile provare ad ottenere cio' che voglio perche' probabilmente non ci riusciro'	○	○

DH Post Covid-19 UNITE!

Valutazione Psichiatrica

Record ID ______________________

Data valutazione ______________________
(Inserire gg-mm-aaaa)

BPRS - BRIEF PSYCHIATRIC RATING SCALE

	NV=non valutato	1= assente	2= molto lieve	3= lieve	4= moderato	5= moderatamente grave	6= grave	7= molto grave
1) PREOCCUPAZIONI SOMATICHE	○	○	○	○	○	○	○	○
2) ANSIA	○	○	○	○	○	○	○	○
3) DEPRESSIONE	○	○	○	○	○	○	○	○
4) RISCHIO DI SUICIDIO	○	○	○	○	○	○	○	○
5) SENTIMENTI DI COLPA	○	○	○	○	○	○	○	○
6) OSTILITA'	○	○	○	○	○	○	○	○
7) ELEVAZIONE DEL TONO DELL'UMORE	○	○	○	○	○	○	○	○
8) GRANDIOSITA'	○	○	○	○	○	○	○	○
9) SOSPETTOSITA'	○	○	○	○	○	○	○	○
10) ALLUCINAZIONI	○	○	○	○	○	○	○	○
11) CONTENUTO INSOLITO DEL PENSIERO	○	○	○	○	○	○	○	○
12) COMPORTAMENTO BIZZARRO	○	○	○	○	○	○	○	○
13) TRASCURATEZZA DI SE	○	○	○	○	○	○	○	○
14) DISORIENTAMENTO	○	○	○	○	○	○	○	○
15) DISORGANIZZAZIONE CONCETTUALE	○	○	○	○	○	○	○	○
16) APPIATTIMENTO AFFETTIVO	○	○	○	○	○	○	○	○
17) ISOLAMENTO EMOTIVO	○	○	○	○	○	○	○	○
18) RALLENTAMENTO MOTORIO	○	○	○	○	○	○	○	○
19) TENSIONE MOTORIA	○	○	○	○	○	○	○	○
20) MANCANZA DI COOPERAZIONE	○	○	○	○	○	○	○	○
21) ECCITAMENTO	○	○	○	○	○	○	○	○
22) DISTRAIBILITA'	○	○	○	○	○	○	○	○
23) IPERATTIVITA' MOTORIA	○	○	○	○	○	○	○	○
24) MANIERISMO E POSTURE	○	○	○	○	○	○	○	○

HAM-A	0= assente	1= lieve	2= moderato	3= grave	4= molto grave
1) Ansia	○	○	○	○	○
2) Tensione	○	○	○	○	○
3) Paure	○	○	○	○	○
4) Insonnia	○	○	○	○	○
5) Sfera intellettiva	○	○	○	○	○
6) Umore depresso	○	○	○	○	○
7) Sintomi somatici (apparato muscolare) ○	○	○	○	○	
8) Sintomi somatici (organi di senso) ○	○	○	○	○	
9) Sintomi cardiovascolari	○	○	○	○	○
10) Sintomi respiratori	○	○	○	○	○
11) Sintomi gastrointestinali	○	○	○	○	○
12) Sintomi genito-urinari	○	○	○	○	○
13) Sintomi a carico del SN	○	○	○	○	○
14)Comportamento del soggetto durante l'esame ○	○	○	○	○	

HAM-D	
HAM_D<br>UMORE DEPRESSO	○ Assente<br>○ Manifesta questi sentimenti solo se interrogato<br>○ Esprime spontaneamente questi sentimenti<br>○ Comunica questi sentimenti con messaggi non verbali, cioe' attraverso l'espressione del volto, la posizione del corpo, la voce e la tendenza al pianto<br>○ Il paziente manifesta quasi esclusivamente questi sentimenti mediante messaggi sia verbali che non verbali
HAM_D<br>SENTIMENTI DI COLPA	○ Assenti<br>○ Autoaccusa, pensa di aver deluso la gente<br>○ Idee di colpa o ripensamenti su errori passati o su azioni peccaminose<br>○ L'attuale malattia e' una punizione. Deliri di colpa<br>○ Ode voci di accusa o di denigrazione e/o ha esperienze allucinatorie visive a contenuto minaccioso
HAM_D<br>SUICIDIO	○ Assente<br>○ Pensa che la vita non valga la pena di essere vissuta<br>○ Vorrebbe essere morto o pensa alla possibilita' di suicidarsi<br>○ Idee o gesti di suicidio<br>○ Tentativi di suicidio (ogni serio tentativo deve essere valutato)

HAM_D<br>INSONNIA INIZIALE	◯ Nessuna difficolta' ad addormentarsi<br>◯ Lamenta di avere talvolta difficolta' ad addormentarsi (per es. gli occorre piu' di mezz'ora)<br>◯ Ha sempre difficolta' ad addormentarsi
HAM_D<br>INSONNIA CENTRALE	◯ Nessuna difficolta'<br>◯ Lamenta di essere diventato irrequieto durante la notte<br>◯ Si sveglia durante la notte (segnare 2 ogni volta che si alza dal letto, a meno che non sia per urinare)
HAM_D<br>INSONNIA RITARDATA	◯ Nessuna difficolta'<br>◯ Si risveglia precocemente (nelle prime ore del mattino) ma si riaddormenta<br>◯ e' incapace di riaddormentarsi se si alza dal letto
HAM_D<br>LAVORO E INTERESSI	◯ Nessuna difficolta'<br>◯ Pensieri e sentimenti di incapacita', facile affaticabilita' e debolezza nelle attivita' (lavoro e hobbies)<br>◯ Perdita di interesse per le attivita' - lavoro o hobbies - sia riferite direttamente dal paziente, sia espresse mediante atteggiamenti, quali indifferenza, indecisione ed incertezza (sente che deve sforzarsi di lavorare)<br>◯ Dedica un minor tempo alle attivita' o e' meno efficiente<br>◯ Ha cessato di lavorare a causa della malattia
HAM_D<br>RALLENTAMENTO	◯ Pensiero e linguaggio normali<br>◯ Lieve rallentamento durante il colloquio<br>◯ Evidente rallentamento durante il colloquio<br>◯ Colloquio difficile<br>◯ Stato di arresto psicomotorio
HAM_D<br>AGITAZIONE	◯ Assente<br>◯ Irrequietezza<br>◯ Gioca con le mani, con i capelli ecc.<br>◯ Si muove in continuazione, non riesce a stare seduto<br>◯ Si torce le mani, si morde le unghie, si tira i capelli, si morde le labbra
HAM_D<br>ANSIA PSICHICA	◯ Assente<br>◯ Tensione soggettiva ed irritabilita'<br>◯ Preoccupazioni per questioni di poco conto<br>◯ Atteggiamento apprensivo evidente dalla mimica o dal linguaggio<br>◯ Manifesta spontaneamente paure
HAM_D<br>ANSIA SOMATICA	◯ Assente<br>◯ Lieve<br>◯ Moderata<br>Notevole<br>◯ Invalidante<br>◯

HAM_D<br>SINTOMI SOMATICI GASTROINTESTINALI	◯ Assenti<br>◯ Perdita dell'appetito, ma si alimenta senza essere stimolato o aiutato dal personale. Senso di peso all'addome.<br>◯ Difficolta' ad alimentarsi senza lo stimolo o l'aiuto del personale. Richiede o ha bisogno di lassativi o di farmaci per i disturbi gastrointestinali
HAM_D<br>SINTOMI SOMATICI GENERALI	◯ Assenti<br>◯ Pesantezza agli arti, alla schiena o alla testa. Mal di testa, mal di schiena, dolori muscolari. Perdita di energia e facile affaticabilita'<br>◯ Se i sintomi sono molto evidenti segnare 2
HAM_D<br>SINTOMI GENITALI	◯ Assente<br>◯ Lieve<br>◯ Gravi
HAM_D<br>IPOCONDRIA	◯ Assente<br>◯ Polarizzazione sul proprio corpo<br>◯ Preoccupazione per la propria salute<br>◯ Frequenti lamentele, richieste di aiuto ecc…<br>◯ Deliri ipocondriaci (ferma convinzione di avere una malattia somatica, senza che ve ne siano i motivi)
HAM_D<br>PERDITA DI PESO (SECONDO IL PAZIENTE O I FAMILIARI o VALUTAZIONE OBIETTIVA ESEGUITA PERIODICAMENTE)	◯ Nessuna perdita di peso / meno di mezzo kg in una settimana<br>◯ Probabile perdita di peso associata alla presente malattia / piu' di mezzo kg in una settimana<br>◯ Evidente perdita di peso / piu' di 1 kg in una settimana<br>◯ Non valutata
HAM_D<br>INSIGHT	◯ Non ammalato / Riconosce di essere depresso e ammalato<br>◯ Riconosce di essere ammalato ma attribuisce la responsabilita' alla cattiva alimentazione, al clima, al superlavoro, a malattie infettive, al bisogno di riposo<br>◯ Nega decisamente di essere ammalato
**MRS**	
MRS - MANIA RATING SCALE<br><br>1. INNALZAMENTO DELL'UMORE	◯ Assente<br>◯ Lievemente o probabilmente aumentato su richiesta<br>◯ Innalzamento soggettivo certo, il paziente e' ottimista, sicuro di se', allegro, adeguato al contesto<br>◯ Elevato, inadeguato al contesto, spiritoso<br>◯ Euforico, ride inadeguatamente, canta

MRS - MANIA RATING SCALE<br><br>2. AUMENTO ATTIVITA'-ENERGIA MOTORIA	○ Assente<br>○ Soggettivamente aumentata<br>○ Vivace, gestualita' aumentata<br>○ Energia eccessiva, a volte iperattivo, irrequieto (puo' essere calmato)<br>○ Eccitamento motorio, iperattivita' continua (non puo' essere calmato)
MRS - MANIA RATING SCALE<br><br>3. INTERESSE SESSUALE	○ Normale, non aumentato<br>○ Lievemente o probabilmente aumentato<br>○ Aumento soggettivo certo dietro richiesta<br>○ Soddisfazione sessuale istintiva, elabora storie sessuali, si presenta da ipersessuale<br>○ Comportamenti sessuali manifesti (verso pazienti, personale o intervistatori)
MRS - MANIA RATING SCALE<br><br>4. SONNO	○ Non riferisce riduzione del sonno<br>○ Sonno inferiore al normale fino ad un'ora<br>○ Sonno inferiore al normale oltre un'ora<br>○ Riferisce diminuito il bisogno di dormire<br>○ Nega il bisogno del sonno
MRS - MANIA RATING SCALE<br><br>5. IRRITABILITA'	○ Assente<br>○ Soggettivamente aumentata<br>○ Irritabile talvolta durante l'intervista, recenti episodi di rabbia o di disturbo del reparto<br>○ Frequentemente irritabile durante l'intervista; conciso, risoluto in tutto<br>○ Ostile, non cooperativo; intervista impossibile
MRS - MANIA RATING SCALE<br><br>6. ELOQUIO (ritmo e produttivita')	○ Nessun aumento<br>○ E' loquace<br>○ Aumentati, talvolta, ritmo e produttivita', talvolta prolisso<br>○ Aggressivita' costantemente aumentati ritmo e produttivita', difficile l'interruzione<br>○ Eloquio pressante, ininterrotto, continuo
MRS - MANIA RATING SCALE<br><br>7. DISTURBO DEL LINGUAGGIO-PENSIERO	○ Assente<br>○ Circostanziale, lieve distraibilita', pensieri accelerati<br>○ Distraibile, perde la finalita' del ragionamento, cambia frequentemente gli argomenti, corsa dei pensieri<br>○ Fuga delle idee, tangenzialita', difficolta' a seguire, assonanza, ecolalia<br>○ Incoerenza, comunicazione impossibile
MRS - MANIA RATING SCALE<br><br>8. CONTENUTO	○ Normale<br>○ Progetti incerti, nuovi interessi<br>○ Progetti speciali, iperreligioso<br>○ Idee grandiose o paranoidee, idee di riferimento<br>○ Deliri, allucinazioni
MRS - MANIA RATING SCALE<br><br>9. COMPORTAMENTO AGGRESSIVO-DISTRUTTIVO	○ Assente, cooperativo<br>○ Sarcastico, talvolta chiassoso, sospettoso<br>○ Esigente, fa minacce in reparto<br>○ Minaccia l'intervistatore, grida, intervista difficile<br>○ Aggredisce, e' distruttivo, intervista impossibile

MRS - MANIA RATING SCALE<br><br>10. ASPETTO	◯ Abbigliamento adeguato e curato<br>◯ Minimamente trascurato<br>◯ Scarsamente curato, moderatamente disordinato, abbigliamento vistoso<br>◯ Disordinato, parzialmente vestito, trucco vistoso<br>◯ Completamente trascurato, imbellettato, abbigliamento bizzarro
MRS - MANIA RATING SCALE<br><br>11. CONSAPEVOLEZZA	◯ Non ammalato / Presente, ammette la malattia, in accordo con la necessita' di una terapia<br>◯ Probabilmente ammalato<br>◯ Ammette un cambiamento del comportamento, ma neg la malattia<br>◯ Ammette un possibile cambiamento del comportamento, ma nega la malattia<br>◯ Nega qualsiasi cambiamento del comportamento

**KMDRS**	
Koukopoulos Mixed Depression Rating Scale<br><br>1) ESPRESSIONE DI SOFFERENZA	◯ espressioni laconiche di sofferenza depressiva<br>◯ descrizioni di sofferenza animate e prolungate<br>◯ drammatiche espressioni di sofferenza e disperazione<br>◯ scoppi di lamentele e attacchi di pianto
KOUKOPOULOS MIXED DEPRESSION RATING SCALE<br><br>2) MIMICA FACIALE VIVACE	◯ espressioni faciali ridotte<br>◯ espressioni faciali che manifestano chiaramente le emozioni<br>◯ vivaci espressioni delle emozioni<br>◯ drammatiche espressioni delle emozioni
KOUKOPOULOS MIXED DEPRESSION RATING SCALE<br><br>3) PRODUTTIVITA' DELL'ELOQUIO	◯ eloquio rallentato<br>◯ eloquio nella norma<br>◯ eloquio fluido, conversazione non compromessa<br>◯ chiaramente logorroico, la conversazione e' compromessa
KOUKOPOULOS MIXED DEPRESSION RATING SCALE<br><br>4) LABILITA' EMOTIVA	◯ assente<br>◯ cambiamenti d'umore limitati allo spettro depressivo-disforico, es. passa dalla tristezza alla rabbia<br>◯ cambiamenti sempre all'interno dello spettro depressivo-disforico ma le emozioni sono piu' intense, es. disperazione, furia<br>◯ passa dall'umore depresso all'esaltazione
KOUKOPOULOS MIXED DEPRESSION RATING SCALE<br><br>5) ATTIVITA' MOTORIA/IRREQUIETEZZA<br>Accertarsi che l'iperattivita' motoria/irrequietezza sia visibile (o effettivamente accaduta) e non sia solamente una sensazione soggettiva (e' stato cosi irrequieto e smanioso da non riuscire a rimanere seduto? Deve continuare a camminare su e giu'?)	◯ (0) rallentamento psicomotorio<br>◯ (2) psicomotricita' nella norma<br>◯ (4) evidente irrequietezza ma in grado di rimanere seduto<br>◯ (6) incapace di rimanere seduto o cammina molto spesso su e giu'

KOUKOPOULOS MIXED DEPRESSION RATING SCALE<br><br>6) SENSAZIONI SOGGETTIVE DI IRRITABILITA' E RABBIA NON PROVOCATA	○ assente<br>○ lamenta irritabilita' per ragioni minori<br>○ si sente abbastanza arrabbiato/a senza alcuna ragione<br>○ sente la voglia di rompere delle cose (o sente la voglia di agire contro se stesso/a o altri)
KOUKOPOULOS MIXED DEPRESSION RATING SCALE<br>7) MANIFESTE ESPRESSIONI DI IRRITABILITA' E RABBIA<br>(Come ha manifestato la sua rabbia, insofferenza, irritabilita'? Ha avuto discussioni? Ha perso la pazienza, ha lanciato o rotto oggetti? Le e' successo di colpire fisicamente qualcuno?)	○ (0) assente<br>○ (2) manifesta facilmente insofferenza, impazienza, occasionalmente aggressivo/a verbalmente<br>○ (4) occasionalmente violento/a contro gli oggetti<br>○ (6) occasionalmente violento/a contro se stesso o altri
KOUKOPOULOS MIXED DEPRESSION RATING SCALE<br>8) ACCELERAZIONE E/O AFFOLLAMENTO DEI PENSIERI<br><br>Notare i riferimenti spontanei prima di porre la domanda diretta.<br>(Ha avuto piu' pensieri del solito o piu' di quanti ne riesca a gestire? Ha interferito con il suo prendere sonno? I suoi pensieri si sono rincorsi velocemente?)	○ assente<br>○ ruminazioni depressive o dolorose<br>○ pensieri o ricordi affollati, alcuni dei quali potrebbero non essere dolorosi.<br>○ pensieri accelerati o motivi musicali persistenti nella testa.
KOUKOPOULOS MIXED DEPRESSION RATING SCALE<br>9) TENSIONE INTERNA<br><br>Notare i riferimenti spontanei prima di porre la domanda diretta. Questa e' una sensazione particolare e stressante di tensione nervosa. Questa sensazione puo' essere accompagnata dalla sensazione di essere bloccati nelle attivita' mentali. (Si sente con i nervi a fior di pelle, teso/a o stressato/a? Questa tensione le impedisce di fare le cose, di pensare?)	○ assente<br>○ questa sensazione e' riportata solo se elicitata<br>○ questa sensazione e' riportata spontaneamente come una parte stressante della condizione<br>○ il/la paziente riporta di essere tormentato/a da una sensazione di tensione e agitazione interna. La pressione arteriosa diastolica puo' essere > 90 mmHg.
KOUKOPOULOS MIXED DEPRESSION RATING SCALE<br>10) TENSIONE MUSCOLARE<br><br>Ha avuto difficolta' a rilassare i muscoli da quando e' depresso/a? Sente i suoi muscoli tesi? Dove ha sentito la tensione? Era in grado di rilassarsi?)	○ assente<br>○ tensione muscolare riportata solo in seguito a domanda<br>○ tensione muscolare lieve e ricorrente con qualche possibilita' di rilassarsi<br>○ definita esperienza di tensione muscolare senza capacita' di rilassarsi, o tensione visibile (possibili tremori). Il paziente puo' lamentare dolenzia muscolare, soprattutto al collo e alla schiena.
KOUKOPOULOS MIXED DEPRESSION RATING SCALE<br>11) INSONNIA INIZIALE E CENTRALE<br><br>(Ha avuto difficolta' ad addormentarsi? Quanto tempo impiega ad addormentarsi una volta andato a letto? Si sveglia nel mezzo della notte? Se si sveglia, riesce a riaddormentarsi?	○ assente<br>○ lamenta difficolta' di addormentamento della durata di non piu' di mezz'ora<br>○ lamenta difficolta' di addormentamento della durata di piu' di un'ora e/o due o piu' risvegli durante la notte<br>○ insonnia pressoche' totale per la difficolta' di addormentamento e per i numerosi risvegli durante la notte

KOUKOPOULOS MIXED DEPRESSION RATING SCALE<br><br>12) IMPULSIVITA' SUICIDA<br><br>(Ha avuto pensieri di uccidersi o di farsi del mal? Questi pensieri erano impulsivi, arrivavano all'improvviso? Spesso in momenti di rabbia? Ha mai effettivamente fatto qualcosa?)	○ (0) assenti<br>○ (2) pensieri suicidi possibilmente impulsivi<br>○ (4) pensieri suicidi impulsivi certamente presenti<br>○ (6) tentativo/i di suicidio impulsivi (come tentare di gettarsi da una macchina)
KOUKOPOULOS MIXED DEPRESSION RATING SCALE<br><br>13) SESSUALITA'<br><br>Quando una persona diviene depressa ha meno desiderio sessuale del solito. Ha notato occasionalmente un aumento del desiderio sessuale e/o dell'attivita'?	○ attivita' e/o desiderio sessuale ridotti<br>○ attivita' sessuale nella norma<br>○ ipersessualita' occasionale<br>○ frequente ipersessualita'
KOUKOPOULOS MIXED DEPRESSION RATING SCALE<br><br>14) SINTOMI PSICOTICI<br><br>Ha mai pensato che gli altri stessero facendo qualcosa contro di lei? Ha mai pensato di essere al centro degli interessi degli altri? Ha mai sentito rumori o voci?	○ assenti<br>○ sospettosita'<br>○ idee di riferimento<br>○ deliri e/o allucinazioni

CAPS - Criterio B. Sintomi intrusivi (need 1 for diagnosis)

	Assente	Lieve	Moderato	Grave	Molto Grave
Ricordi spiacevoli ricorrenti e ○ intrusivi dell'evento	○	○	○	○	
Sogni spiacevoli ricorrenti ○ dell'evento	○	○	○	○	
Reazioni dissociative (ad ○ esempio, flashback) in cui l'individuo si sente o agisce come se l'evento traumatico si stesse ripresentando. (Tali reazioni possono verificarsi su un continuum, con l'espressione piu' estrema di una completa perdita di consapevolezza del presente)	○	○	○	○	
Disagio psicologico intenso ○ all'esposizione a fattori scatenanti interni o esterni che simbolizzano o assomigliano a qualche aspetto dell'evento traumatico	○	○	○	○	

Reattivita' fisiologica o ○ interni o esterni che simbolizzano o assomigliano a qualche aspetto dell'evento traumatico	○	○	○ esposizione a fattori scatenanti	○	

CAPS - Criterio C. Sintomi d'evitamento (need 1 for diagnosis)

	Assente	Lieve	Moderato	Grave	Molto grave
Sforzi per evitare ricordi, ○ pensieri e sensazioni associate con il trauma	○	○	○	○	
Sforzi per evitare attivita', luoghi ○ del trauma	○	○	○ o persone che evocano ricordi	○	

CAPS - Criterio D. Sintomi dell'umore e cognitivi (need 2 for diagnosis)

	Assente	Lieve	Moderato	Grave	Molto grave
Incapacita' di ricordare qualche aspetto importante del trauma	○	○	○	○	○
Credenze negative o aspettative su se stessi, gli altri, o il mondo	○	○	○	○	○
Persistente, distorta cognizione circa la causa o le conseguenze dell'evento traumatico che conducono l'individuo ad incolpare se stesso o gli altri	○	○	○	○	○
Persistente stato emotivo negativo (ad esempio, paura, orrore, rabbia, senso di colpa, o vergogna)	○	○	○	○	○
Notevole diminuzione dell'interesse o della partecipazione ad attivita' significative	○	○	○	○	○
Sentimenti di distacco o di estraneita' verso gli altri	○	○	○	○	○
Persistente incapacita' di sperimentare emozioni positive	○	○	○	○	○

CAPS - Criterio E. Sintomi arousal e reattività (need 2 for diagnosis)	Assente	Lieve	Moderato	Grave	Molto grave
Irritabilita' o scoppi di collera	◯	◯	◯	◯	◯
Comportamento avventato o autodistruttivo	◯	◯	◯	◯	◯
Ipervigilanza	◯	◯	◯	◯	◯
Esagerate risposte di allarme	◯	◯	◯	◯	◯
Problemi di concetrazione	◯	◯	◯	◯	◯
Difficolta' ad addormentarsi o a mantenere il sonno	◯	◯	◯	◯	◯

CAPS - Criterio F. Durata (needed for diagnosis)	
CAPS (Criterio F): La sintomatologia dura da almeno un mese?	◯ No ◯ Si

CAPS - Criterio G. Distress o impairment (needed for diagnosis)	
CAPS (Criterio G) Il disturbo causa un disagio clinicamente significativo o menomazione nel funzionamento sociale, lavorativo o di altre aree importanti?	◯ No ◯ Si

CAPS (Criterio G)	Assente	Lieve	Moderato	Grave	Molto grave
Stress soggettivo	◯	◯	◯	◯	◯
Alterazione del funzionamento sociale	◯	◯	◯	◯	◯
Alterazione del funzionamento lavorativo o un'altra area importante di funzionamento	◯	◯	◯	◯	◯

CAPS<br>26) Validita' globale<br>stimare la validita' complessiva delle risposte. Considerare i fattori<br>come la compliance al colloquio, lo stato mentale (ad es.<br>problemi di concentrazione, comprensione degli oggetti,<br>dissociazione), e la prova di sforzi per esagerare o ridurre al minimo i sintomi	◯ Eccellente, nessun motivo per sospettare risposte non valide<br>◯ Buono, fattori che possono influire negativamente la validita'<br>◯ Equo, fattori presenti che sicuramente riducono la validita'<br>◯ Validita' scadente e sostanzialmente ridotta<br>◯ Risposte non valide, stato mentale gravemente compromesso

CAPS<br>27) Gravita' globale<br>stimare la gravita' complessiva dei sintomi di PTSD. Considerare il grado di disagio soggettivo,il grado dimenomazioni, l'osservazione dei comportamenti durante colloquio e il giudizio per quanto riguarda lo stile di risposta	○ Nessun sintomo clinicamente significativo, nessuna sofferenza e nessun danno funzionale<br>○ Disturbo lieve, minimo o danno funzionale<br>○ Disagio moderato, definito o funzionale compromissione ma funziona in modo soddisfacente con lo sforzo<br>○ Grave, notevole disagio o funzionale menomazione, funzionamento limitato anche con sforzo<br>○ Disturbo estremo, marcato o marcato deterioramento in due o piu' aree principali di funzionamento

CAPS - Sottotipo con sintomi dissociativi (need 1 for subtype)	
CAPS<br>29) Depersonalizzazione: Esperienze persistenti o ricorrenti di sentirsi distaccati, e come se si fosse un osservatore esterno, dei propri processi mentali o del proprio corpo	○ Assente<br>○ Lieve / sottosoglia<br>○ Moderato / soglia<br>○ Grave / notevolmente elevata<br>○ Estremo / inabilitazione
CAPS<br>30) Derealizzazione: esperienze persistenti o ricorrenti di irrealta' dell'ambiente circostante	○ Assente<br>○ Lieve / sottosoglia<br>○ Moderato / soglia<br>○ Grave / notevolmente elevata<br>○ Estremo / inabilitazione
